# Optogenetic Interrogation of Electrophysiological Dendritic Properties and Their Effect on Pacemaking Neurons from Acute Rodent Brain Slices

**DOI:** 10.21769/BioProtoc.4992

**Published:** 2024-05-20

**Authors:** Naomi Gilin, Nadine Wattad, Lior Tiroshi, Joshua A. Goldberg

**Affiliations:** 1 Department of Medical Neurobiology, Institute of Medical Research Israel – Canada, Faculty of Medicine, The Hebrew University of Jerusalem, Jerusalem, Israel; 2 Department of Neurobiology, Northwestern University, Evanston, IL, USA

**Keywords:** Dendrites, Pacemaking neurons, Acute brain slice, Whole-cell patch clamp, Perforated patch, Optogenetics, Phase resetting, Entrainment

## Abstract

Understanding dendritic excitability is essential for a complete and precise characterization of neurons’ input-output relationships. Theoretical and experimental work demonstrates that the electrotonic and nonlinear properties of dendrites can alter the amplitude (e.g., through amplification) and latency of synaptic inputs as viewed in the axosomatic region where spike timing is determined. The gold-standard technique to study dendritic excitability is using dual-patch recordings with a high-resistance electrode used to patch a piece of distal dendrite in addition to a somatic patch electrode. However, this approach is often impractical when distal dendrites are too fine to patch. Therefore, we developed a technique that utilizes the expression of Channelrhodopsin-2 (ChR2) to study dendritic excitability in acute brain slices through the combination of a somatic patch electrode and optogenetic activation. The protocol describes how to prepare acute slices from mice that express ChR2 in specific cell types, and how to use two modes of light stimulation: proximal (which activates the soma and proximal dendrites in a ~100 µm diameter surrounding the soma) with the use of a high-magnification objective and full-field stimulation through a low-magnification objective (which activates the entire somato-dendritic field of the neuron). We use this technique in conjunction with various stimulation protocols to estimate model-based spectral components of dendritic filtering and the impact of dendrites on phase response curves, peri-stimulus time histograms, and entrainment of pacemaking neurons. This technique provides a novel use of optogenetics to study intrinsic dendritic excitability through the use of standard patch-clamp slice physiology.

Key features

• A method for studying the effects of electrotonic and nonlinear dendritic properties on the sub- and suprathreshold responses of pacemaking neurons.

• Combines somatic patch clamp or perforated patch recordings with optogenetic activation in acute brain slices to investigate dendritic linear transformation without patching the dendrite.

• Oscillatory illumination at various frequencies estimates spectral properties of the dendrite using subthreshold voltage-clamp recordings and studies entrainment of pacemakers in current clamp recordings.

• This protocol uses Poisson white noise illumination to estimate dendritic phase response curves and peri-stimulus time histograms.

## Background

Whole-cell voltage and current clamp recordings are the fundamental tools for studying neuronal excitability and synaptic physiology. Using the methodology laid out by Hodgkin & Huxley [1], neurophysiologists measure the membrane currents in voltage-clamp mode to elucidate how they shape the voltage trajectory of a neuron as measured in current-clamp mode. However, unlike studies in primary neuronal cultures [2–5] where neurons can be assumed to be isopotential, studies in acute brain slices include neurons with elaborate and extensive dendritic arbors, whose properties are overlooked. In general, dendritic properties will alter the physiological measurement, particularly when estimating synaptic inputs, where the linear cable properties and the nonlinear conductances in the dendrites reshape the synaptic currents and potentials. One approach to overcome this is to use dual-patch clamp measurements, where the soma and the dendrites are patched simultaneously, allowing for direct interrogation of how the dendrite transforms synaptic voltage perturbations [6–11]. Because synaptic inputs are typically weak, even if the dendrites are endowed with strong nonlinearities (e.g., various voltage-activated currents), the dendritic transformation can be considered a linear transformation. Linear time-invariant (LTI) transformations can be characterized by their impulse response to an instantaneous perturbation in the time domain or by their amplitude and phase responses in the Fourier frequency space [12]. Using the latter approach, one can perturb the dendrite with sinusoidal inputs at various frequencies or with an approximation of white noise that simultaneously elicits (nominally) all frequencies. Indeed, this methodology has been used in dual-patch experiments where the perturbation is introduced via the dendritic electrode and measured via the somatic one [7,8,10,11]. However, such dual-patch recordings are not always possible either because the dendrites are too small to visualize or too small to patch. We therefore developed a method that harnesses optogenetics to interrogate the dendritic LTI transformation using sinusoidal and white noise perturbation. Channelrhodopsin-2 (ChR2) is expressed in the neuronal type being investigated (in our case, we used transgenic mice that express ChR2 in specific cell types). We then visually guide a patch pipette to the soma of one of these neurons in an acute brain slice to record its electrical activity. To characterize the dendritic LTI transformation in the time domain, we use either individual brief pulses, or Poisson barrages of pulses of blue (470 nm) LED light to activate ChR2 currents, as pioneered by Higgs and Wilson [13], or sinusoidally modulated blue LED light. We situate the soma at the center of the objective’s field of view so that all blue light perturbations are applied to a somatodendritic region of the cell surrounding the soma and measure their effect on the soma. This is done either in voltage-clamp mode to measure how the currents are transformed or in current-clamp mode to study how the light perturbations affect the autonomous firing of pacemaking neurons (we study basal ganglia pacemakers [14,15]). We either illuminate the entire field of view via a low-magnification microscope objective or we constrain the localization of the blue light to a small perisomatic region (~100 μm diameter that encompasses the soma even for neurons with large somata) by occluding the blue LED light path via a high-magnification objective. We then compare the results from the full-field illumination with those from the proximal illumination to deduce the contribution to the responses that can be attributed solely to the dendrites. Future applications could use patterned laser stimulation and/or multiphoton activation of the opsins for truly localized dendritic (or somatic) stimulation.

## Materials and reagents

**Biological materials**

1. Mice that express channelrhodopsin-2 in a population of pacemaking neurons. We used either Thy1-ChR2 mice [B6.Cg-Tg(Thy1-COP4/EYFP) 18Gfng/J] (Jackson laboratory, stock: 007612) or ChAT-ChR2 mice: Ai32 mice [RCL-ChR2(H134R)/EYFP] that express flexed ChR2 and an EYFP fusion protein under the CAG promoter (Jackson laboratory, stock: 012569) cross-bred with ChAT-IRES-Cre (∆neo) that express Cre recombinase under the Chat promoter (Jackson laboratory, stock: 031661)

**Reagents**

1. NaCl (Sigma-Aldrich, CAS number: 7647-14-5)
2. KCl (Sigma-Aldrich, CAS number: 7447-40-7)
3. NaH_2_PO_4_·H_2_O (Sigma-Aldrich, CAS number: 10049-21-5)
4. CaCl_2_·2H_2_O (Sigma-Aldrich, CAS number: 10035-04-8)
5. MgSO_4_ anhydrous (Sigma-Aldrich, CAS number: 7487-88-9)
6. NaHCO_3_ (Sigma-Aldrich, CAS number: 144-55-8)
7. Dextrose (Sigma-Aldrich, CAS number: 50-99-7)
8. Sucrose (Sigma-Aldrich, CAS umber: 57-50-1)
9. Ascorbic acid (Sigma-Aldrich, CAS number: 50-81-7)
10. KCH_3_SO_3_ (Sigma-Aldrich, CAS number: 2386-56-3)
11. EGTA (Sigma-Aldrich, CAS number: 67-42-5)
12. HEPES (Sigma-Aldrich, CAS number: 7365-45-9)
13. Na_2_-phosphocreatine (Sigma-Aldrich, CAS number: 19333-65-4)
14. ATP magnesium salt (Sigma-Aldrich, CAS number: 74804-12-9)
15. GTP sodium salt (Sigma-Aldrich, CAS number: 36051-31-7)
16. Tetrodotoxin citrate (HelloBio, CAS number: 18660-81-6)
17. ZD 7288 (MedChemExpress, CAS number: 133059-99-1)
18. Ketamine (as hydrochloride) 1 g/10 mL (Vetoquinol, Clorketam^®^)
19. Xylazine (as hydrochloride) 20 mg/mL (euroVet, Sedaxylan^®^)
20. Gramicidin (Sigma-Aldrich, catalog number: G5002, CAS number: 1405-97-6).
*Note: This is a mixture of Gramicidin A,B,C&D and requires higher concentrations of stock solutions than appear in publications using only Gramicidin B [16].*
21. DNQX disodium salt (HelloBio, catalog number: 2379-57-9)
22. D-AP5 (HelloBio, catalog number: 79055-68-8)
23. SR 95531 hydrobromide (gabazine) (HelloBio, catalog number: 104104-50-9)
24. CGP55845 hydrochloride (HelloBio, catalog number: 149184-22-5)
25. Dimethyl sulfoxide (DMSO), anhydrous (Sigma-Aldrich, catalog number: 276855, CAS number: 67-68-5)

**Solutions**

1. 10× artificial cerebrospinal fluid (aCSF) stock solution (see Recipes)
2. 10× modified aCSF (Sucrose) stock solution (see Recipes)
3. K internal solution (see Recipes)

**Recipes**

1. **10× aCSF stock solution in ddH_2_O (1,000 mL)** *Note: The stock solution is prepared without the sodium bicarbonate. For the final solution, dilute 1:10 in ddH_2_O and add 2.18 g of NaHCO_3_.*
  **Table:** 

Reagent	Final concentration	Quantity or Volume
NaCl	126 mM	73.63 g
KCl	2.5 mM	1.86 g
NaH_2_PO_4_·H_2_O	1.25 mM	1.724 g
CaCl_2_·2H_2_O	2.0 mM	2.94 g
MgSO_4_ anhydrous	2.0 mM	2.408 g
Dextrose	10 mM	18.02 g
NaHCO_3_	26 mM	see note
2. **10× modified aCSF (sucrose) stock solution in ddH_2_O (500 mL)**
*Note: The stock solution is prepared without the sodium bicarbonate. For the final solution, dilute 1:10 in ddH_2_O and add 1.09 g of NaHCO_3_. Sucrose is added (36 g/500 mL) to the final solution. The aCSF is modified to have much lower concentrations of sodium and calcium to prevent excitotoxicity while the tissue is being dissected under hypoxic conditions. (Similarly, the concentration of magnesium is increased in order to prevent NMDA receptors from contributing to excitotoxicity.) The sucrose compensates for the reduced osmolarity in this sodium-poor solution* [17].
  **Table:** 

Reagent	Final concentration	Quantity or Volume
Sucrose	210 mM	see note
KCl	2.5 mM	0.932 g
NaH_2_PO_4_·H_2_O	1.25 mM	0.862 g
CaCl_2_·2H_2_O	0.5 mM	0.37 g
MgSO_4_ anhydrous	10 mM	6.02 g
Dextrose	10 mM	9.01 g
Ascorbic acid	0.4 mM	0.35 g
NaHCO_3_	26 mM	see note
3. **K internal solution (90 mL)**
*Note: All ingredients, except ATP and GTP, are mixed in approximately 75 mL of ice-cold ddH_2_O. Use pH meter to titer to pH 7.3–7.4 with 1 N KOH (using 50 µL increments with a pipettor); then, top off to 90 mL with ice-cold ddH_2_O. Measure osmolarity with an osmometer, which should be 280–300 mOsm/kg. If pH and osmolarity are good, add ATP and GTP, readjust the pH with 1N KOH, rapidly aliquot to 1 mL Eppendorf tubes, and freeze (-20˚C). Do the whole procedure on ice.*
  **Table:** 

Reagent	Final concentration	Quantity or Volume
KCH_3_SO_3_	135.5 mM	1.637 g
EGTA	0.2 mM	6.8 mg
KCl	5 mM	33.5 mg
NaCl	2.5 mM	13.15 mg
Na_2_-phosphocreatine	5 mM [18]	114.8 mg
HEPES	10 mM	238.3 mg
ATP magnesium salt	2.0 mM	100 mg, see note
GTP sodium salt	0.21 mM	10 mg, see note

**Laboratory supplies**

1. Single-edge razor blade (for vibratome) (WPI, catalog number: BLADES-2)
2. Single-edge industrial blades (for blocking brain) (any will do, we use Excel, catalog number: 22609)
3. 1 mL insulin syringe (30 G 5/16” 0.3 × 08 mm) (PiC solution, catalog number: 02 022726 100 150)
4. 25G scalp vein sets (KDL, catalog number: 4193)
5. Super glue (Brush-On) (Loctite)
6. Filter paper (Whatman, catalog number: 1002-070)
7. Dental wax (Electron Microscopy Sciences, catalog number: 72660)
8. Harp (to hold down slice in recording chamber) (Warner Instruments, catalog number: 641418, or other sizes)
9. 50 mL glass cup
10. 250 mL plastic cups
11. 20 mL Luer-lock syringes
12. 4 mm syringe driven 0.22 μm filter units (Millex^®^ GV, catalog number: SLGVR04NL)
13. Microloader for Eppendorf pipettes (Eppendorf, catalog number: 5242 956.003)
14. Needles, 16 G, 19 G, and 27 G
15. 10 mL injectable 0.9% (w/v) saline (NaCl) (Braun 5/12606510/0309)
16. Kimwipes, 4.4 × 8.5 inch (Kimberly-Clark)
17. Borosilicate glass pipettes, with filament (fire polished, length: 10 cm, OD: 1.5 mm, ID: 0.86 mm) (Sutter Instruments, catalog number: BF150-86-10)
18. Microelectrode holder for O.D. 1.5 mm pipettes (Warner Instruments, catalog number: QSW-T15P)

## Equipment

1. Vision Isostation optical workstation (Newport, model: VIS3036-SG4-325A)
2. XY-shifting table + base plate (Luigs & Neumann, catalog number: 200-100 200 0150, 250-200 200 1000)
3. Axioskop microscope (Zeiss) with a “hi-mag” 60×/1.0 NA (FN 26.5) water immersion (WI) objective (Olympus, model: LUMPLFLN60XW) and a “lo-mag” 5×/0.1 NA (FN 22) air/dry objective (Olympus, model: MPLN5). The filter slider was fitted with a dichroic mirror that reflects the 470 nm illumination onto the slice (Chroma, catalog number: T660lpxrxt)
4. Micromanipulator including mounting clamp for headstage (Luigs & Neumann, catalog number: 210-100 000 0010)
5. Slice chamber (Luigs & Neumann, catalog number: 200-100 500 0150)
6. Temperature controller (Luigs & Neumann, catalog number: 200-100 500 0145)
7. Remote control system for XY-shifting table and micromanipulator (Luigs & Neumann, catalog number: 200-100 900 9050, 200-100 900 7911)
8. Amplifier and headstage (Molecular Devices, model: MultiClamp 700B)“
9. A/D board (CED, model: Power 1401-3)
10. 470 nm LED and analog driver (Mightex, model: LCS-0470-03-22, SLA-1000-2)
11. Monochrome video camera (iDS, model: UI-1240LE-NIR-GL)
12. Micropipette puller (Sutter, model: P-1000)
13. Vibratome (Leica, model: VT1000 S)
14. Osmometer (Advanced Instruments, model: 3320)
15. Manometer (Dwyer, model: Series 475 mark III)
16. Hand blender (any that is appropriate for crushing ice)
17. Surgical scissors sharp-blunt (for decapitation) (FST, catalog number: 14001-14)
18. Fine scissors sharp (for cutting skull bone) (FST, catalog number: 14060-09)
19. Vannas fine spring scissors (for cutting right atrium) (Roboz, catalog number: RS-5620)
20. Bracken forceps (Roboz, catalog number: RS-5211)
21. Carbogen (95% O_2_ + 5% CO_2_) supply tank
22. Lab microspatulas with various ends

## Software and datasets

1. Signal 6.05a (CED, 2/14/2018), licensed)
2. Multiclamp commander (Molecular devices, 2018, licensed)
3. uEye Cockpit 4.94.000 (iDS, 2020)
4. MATLAB R2020b update 2 (Mathworks, 11/3/2020, licensed)

## Procedure

- **Acute brain slice preparation**
  1. Prepare K internal, 10× sucrose, and 10× aCSF at least one day in advance (K internal is good for approximately two months, 10× aCSF for one month, and sucrose solution for approximately two weeks). Dilute the 10× sucrose solution to the final solution (500 mL) and add bicarbonate and sucrose. Mix the solution with a magnetic stirrer, then check its osmolarity with an osmometer. The osmolarity should be 290–310 mOsm. Oxygenate the solution for at least 15 min and freeze 120 mL (-20 °C) in a 250 mL plastic cup for using on one experiment day. Refrigerate the remainder of sucrose and aCSF solutions.
  2. On the day of dissection, prepare equipment and final solutions:
    - Thaw the frozen sucrose solution (e.g., by submerging the plastic cup with frozen solution in a container with warm tap water of any kind).
*Note: Make sure none of the tap water mixes into the sucrose solution. Let the sucrose thaw a bit and, when possible, insert a carbogen tube into the mixture. While the sucrose continues to thaw, perform the following steps.*
    - Dilute the 10× aCSF solution to the final solution (500 mL), add bicarbonate, and mix (this time the solution can be mixed manually due to the small amount of solute). Check the solution’s osmolarity, which should be 290–310 mOsm. Pour enough of the solution into a slice chamber so that the slices are submerged and insert a carbogen tube into the aCSF. Make sure that there are no air bubbles in the chamber, since they might prevent aCSF solution from reaching brain slices and causing cell death.
**Critical: Avoid moving the chamber from now on to prevent air bubble formation.**
    - Fill two large containers with ice. In one (container 1), secure a Styrofoam block (perfusion board) in the ice, preferably tilted downwards on one side. Place all surgical tools in the container so that the parts meant to come in contact with the mouse’s brain are covered in ice (Figure 1A). In the other container (container 2), place a single-edge industrial blade, vibratome plate, and dental wax sheet.
**Critical: The vibratome plate should be set on Kimwipes on the ice so that ice does not stick to its bottom when you are later ready to insert the slide into the vibratome.**
      **Figure 1.**
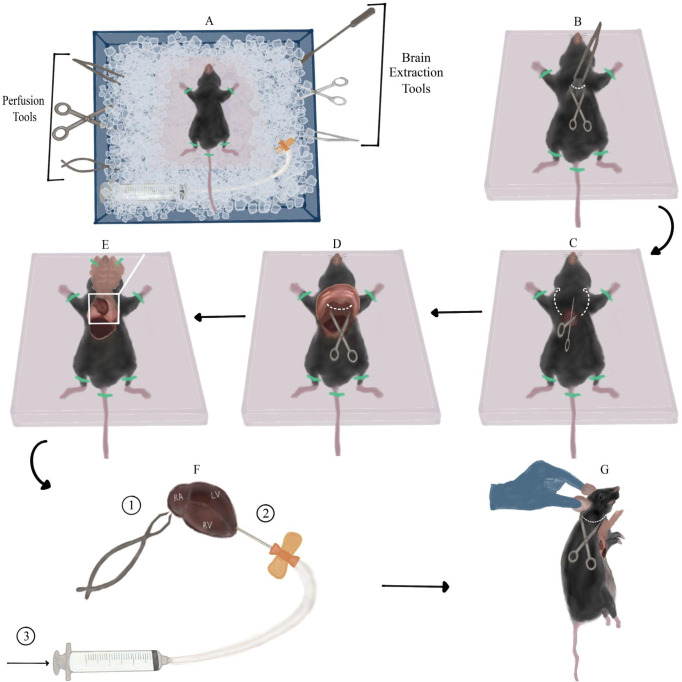
Mouse dissection and perfusion with sucrose. A. Overview of the dissection station arrangement (top view): ice container, surgery tools, and mouse taped to Styrofoam board. Note the Styrofoam board tilted downwards, and surgery tools submerged in ice. B. First incision: lifting the skin using Bracken forceps and creating a transverse incision across the raised piece of skin (along the white dotted line) using sharp-blunt dissecting scissors. C. Detaching the rib cage: inserting the scissors into the orifice created by the previous step and cutting through both sides of the rib cage (along the dotted arrows). D. Cutting the diaphragm (across dotted line) exposed after lifting the rib cage with forceps. E. View of the exposed thoracic cavity after taping the rib cage to the Styrofoam board. The heart along with the lungs can be seen in the upper part of the aperture, and part of the liver can be seen in the left lower part of the aperture. F. Perfusion steps presented on top of a magnified view of the heart from panel E. First, cut the right atrium (marked RA) with fine spring scissors. Next, insert the scalp vein set needle (connected to the Luer-lock syringe with liquid sucrose) into the left ventricle (marked LV) in the apex area, close to the border with the right ventricle (marked RV). Finally, slowly inject sucrose. G. Detaching the mouse’s head: suspending the mouse by its ears and making a transverse incision under the head (along the dotted line).
    - Prepare a brain slicing station: place the vibratome chamber, filter paper, and super glue in proximity to each other. Insert a new single-edge razor blade into the vibratome’s blade holder and secure it with its finger-tightening screw to connect later to the vibratome head.
**Caution: Position the holder so that the blade is directed away from your hands**.
    - Prepare an anesthesia station: bring the mouse to the dissection area in a cage/cardboard box. While the mouse acclimatizes to its new environment, arrange a surface for mouse fixation and intraperitoneal (i.p.) injection (an Eppendorf tube rack holder, for example). Dilute 1 mL of ketamine and 0.5 mL of xylazine in 8.5 mL of injectable saline (ketamine-xylazine cocktail) and inject 20 μL/g of the cocktail i.p. into the mouse (or slightly more, e.g., for a 20 g mouse, inject at least 0.4 mL cocktail). The dosage per mouse is 200 μg/g ketamine and 20 μg/mg xylazine, or slightly higher, which are terminal dosages.
**Critical: Although the cocktail’s dosage is meant to be terminal, be wary of injecting a significantly larger volume than the minimal volume required, since it might cause premature death of the mouse, before the dissection is ready to begin.** Lay the syringe so that its needle does not come into contact with any surface and points away from you. Do not recap the insulin syringe!
    - Finish preparing the sucrose: now that the sucrose block had time to thaw, there should be an accumulation of liquid sucrose around the frozen chunk. Fill the 20 mL Luer-lock syringe with liquid sucrose, release air bubbles if necessary, and connect it to a scalp vein set. Release a small amount of liquid from the syringe without removing the needle protector to ensure proper connection of the set and removal of excess bubbles to prevent an air embolism while perfusing. Submerge the syringe (connected to the scalp vein set) into container 1, making sure it is completely covered in ice (Figure 1A). **Note: This step should be performed as close as possible to the dissection since from this point on the sucrose in the syringe is no longer oxygenated.** Flip the frozen sucrose block so the part that was submerged in liquid sucrose is now facing upwards so that it will be easier to crush. Using the flat square end of a lab microspatula, crush the frozen sucrose until the mixture is of *slushy-like* consistency. Use a hand blender to crush remaining pieces of frozen sucrose. **Note: It is important that the mixture used for brain slicing is homogenous, since large pieces of frozen sucrose may get in the way of the vibratome’s razor blade.** Pour 40 mL of slushy sucrose into a 50 mL glass cup. Submerge both cups with slushy sucrose in container 2 and insert carbogen tubes into both of them so that the mixtures continue to oxygenate until used.
  3. Dissection of the mouse:
    - Inject the ketamine-xylazine cocktail i.p. and return the mouse immediately back to the cage/box. The first sign of mouse’s insensibility is recumbency (motionless head and body, loss of muscle tone) [19], which will appear in a matter of seconds to minutes, depending on the mouse’s weight and anesthetic cocktail dosage. Monitor the mouse and pay close attention to its breathing rate, which should gradually decrease.
    - Once the mouse’s breathing rate has significantly decreased (<55 breaths/min) [20], transfer it to the perfusion board and lay it on its back. Prior to initiating the procedure, evaluate anesthetic depth by testing the pedal withdrawal reflex: apply alternating hind paw toe pinches every 10 s between the metatarsal and phalanges bone. Appropriate anesthetic depth is determined by the absence of a pain response, measured by three consecutive non-responses to alternating pinches (the mouse does not twitch and withdraw its paws) [19]. Procedure may begin only after the pedal withdrawal reflex disappears. In case pain response persists after multiple tests, inject i.p. up to another third of the initial ketamine-xylazine dosage and repeat the testing process. Procedure should begin when the mouse has no pain response but is still breathing (very slowly).
    - Stretch the mouse’s limbs to the sides and tape them to the perfusion board with adhesive tape to create a smooth, taut, and fixed surface for dissection. Using Bracken forceps, elevate a large piece of the mouse’s skin and fur around the tip of the sternum (the xiphoid process appears as a slight protuberance between the two halves of the rib cage) (Figure 1B). Using sharp-blunt dissecting scissors, create a transverse incision across the raised piece of skin. Repeat the same step on the uncovered fascia below the skin. These two incisions should create a small round orifice (Figure 1B).
    - Insert the scissors vertically into the orifice with the sharp edge facing upwards (this will prevent damage to internal organs). Cut once through both sides of the rib cage by sliding the scissors all the way from the orifice to the base of the neck. These cuts should loosen the rib cage, leaving the diaphragm as a holding point (Figure 1C). Lift the rib cage with Bracken forceps and cut the diaphragm using the same dissection scissors (Figure 1D). Now that the chest flap is detached, flip it on its longitudinal axis towards the neck and tape it to the perfusion board/mouse’s body with adhesive tape. The thoracic cavity should now be exposed and the heart visible (Figure 1E).
    - Grab the heart with Bracken forceps and pull it up for better visibility. Using fine spring scissors, cut the right atrium (Figure 1F, 1), causing pooling of blood around the heart. Next, remove the needle protector from the scalp vein set and insert the needle vertically into the left ventricle in proximity to the apex (Figure 1F, 2), while simultaneously firmly holding the heart with Bracken forceps. The needle should enter the ventricle slightly, without puncturing it or entering other cavities of the heart. Without releasing the scalp vein set, use your other hand to slowly inject the sucrose solution into the left ventricle (Figure 1F, 3).
      **Figure 2.**
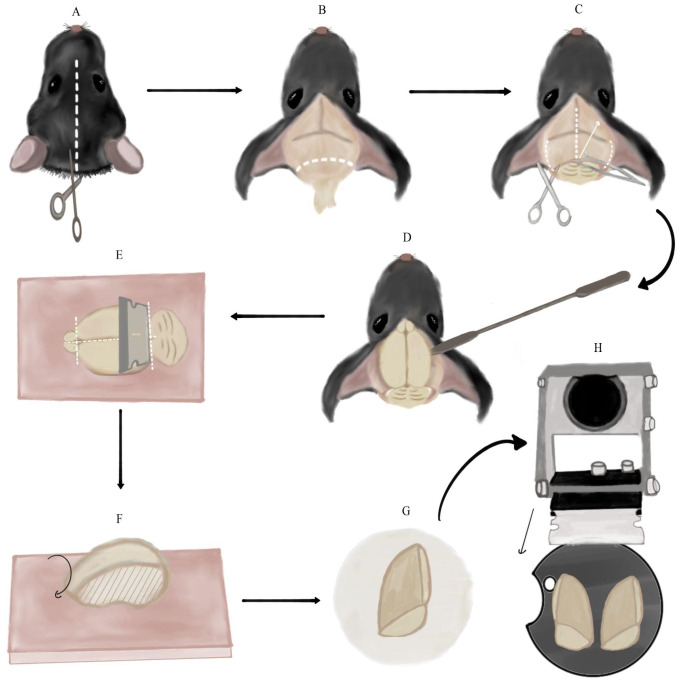
Brain extraction and preparation for slicing. A. Cutting a longitudinal incision in the mouse’s skin from between the ears to the mouse’s eyes (along white dotted line). B. Removing the remaining spinal cord and excess fat and skin by cutting along the dotted line. Note the skin flaps pulled to the sides of the skull. C. Making three incisions in the skull using medium straight, sharp-tipped scissors along the dotted lines. Then, lifting the skull flap using Bracken forceps in the direction of the arrow. D. Detaching the brain from the skull by pushing it with a spatula with an arrow head. E. Separating the cerebellum and olfactory bulbs from the cerebrum using the single-edged industrial blade. Then, severing the cerebral hemispheres using the same blade. All incisions are marked with dotted lines. These steps are performed on the dental wax sheet (pink square). F. Side-view of a single cerebral hemisphere on the dental wax sheet after the previous step. Flipping the hemisphere onto its medial side (marked with stripes). G. Transferring the hemisphere to the filter paper on its medial side (the anterior part of the hemisphere is at the top of the panel). H. Final orientation of the cerebral hemispheres, glued to the vibratome plate, relative to the vibratome blade. The black arrow marks the plane and movement direction of the blade.
    - The flow of sucrose will begin washing out blood from the mouse’s vascular system. The tilted perfusion board will prevent blood aggregation and cause it to flow towards the ice underneath. Escaping blood and liver should transition from being dark-maroon to light-orange as blood is diluted by sucrose. Perfusion should continue until the escaping blood is nearly colorless.
*Note: If blood does not escape to the perfusion board, or it does not get lighter as time goes by, it means that the needle was not inserted deep enough into the left ventricle, or the ventricle was punctured. Conversely, if sucrose solution begins to come out of the mouse’s nose or mouth, it means that the needle was inserted too deeply and entered the right ventricle/left atrium. In both cases, slightly change the position of the scalp vein set accordingly and try to inject the sucrose once again. Note: Perfusing additional cold sucrose solution after the blood clears is also okay and may serve to further cool and protect the brain tissue.*
    - Remove the adhesive tape from the mouse’s limbs and chest flap. Hold the mouse by its head/ears, allowing the rest of its body to be pulled down, and elongate the neck. Using the dissection scissors, make a transverse incision along the neck, right under the head, ideally detaching the entire head with one cut (Figure 1G).
    - Hold the mouse’s head firmly in one hand and create a longitudinal incision in the mouse’s skin from between the ears to the mouse’s eyes (Figure 2A). Separate the pieces of skin and pull them to the sides of the skull. Continue holding the head firmly by the skull (not the skin flaps) and cut the remaining spinal cord along with excess fat and skin, which may be an obstacle to accessing the brain (Figure 2B).
    - Vertically insert medium straight, sharp-tipped scissors between the skull and the brain. Slide the scissors along the longitudinal fissure whilst pushing them against the skull to avoid injuring the brain. Once the scissors reach the area between the eyes, make one incision. Slide the scissors out, continuously pushing them upwards against the skull. Next, insert the scissors horizontally on one side of the skull between the bone and brain. In a similar fashion, insert the scissors **slightly** while pushing them against the inside of the skull and make an incision. Repeat the same step on the other side (Figure 2C).
    - Using Bracken forceps, grab the tip of one skull flap, located in the middle of the ear line close to the longitudinal fissure. Pull it upwards and to the side gently to separate it from the brain (the pulling motion should be in the posteromedial to anterolateral direction). Repeat the same process on the contralateral skull flap (Figure 2C).
*Note: The skull tissue is very thin, and there is a high chance of grabbing brain tissue along with it. Thus, it is advised to remove the flaps very slowly and consecutively check that the brain tissue is untouched.*
    - Insert a spatula with an arrow end under the brain from the side of the skull and slide it to the front of the skull close to the eye line (Figure 2D). Detach the brain from the skull by gently pushing it upwards and drop it into the 50 mL glass cup with sucrose.
    - Wipe the vibratome plate with a Kimwipe, bring it to the brain slicing station, and brush super glue on it.
    - Using a spatula with a spoon end, scoop the brain out of the sucrose and transfer it to a dental wax sheet on its ventral side. **Note: This and the following step describe how to slice sagittal slices of the brain that we use both for striatal and nigral recordings. However, if you are studying another part of the brain, you may block and slice it differently. Nevertheless, some of the principles and pitfalls we describe could still be useful.** Hold the single-edge industrial blade horizontally (vertical to the brain) and cut once along the posterior fissure separating the cerebrum from the cerebellum. Use the same blade to remove the olfactory bulbs from the cerebrum. Next, perform a similar cut along the midline, thus severing the cerebral hemispheres (dashed lines in Figure 2E). **Note: Make sure to perform the cuts as straight as possible and not at an angle.** Use a spatula with an arrow or flat square end to separate brain parts after cutting, if necessary.
    - Flip the hemispheres onto their medial side on the dental wax (Figure 2F) and load each one individually to a flat square spatula. Use the spatula to place the hemispheres on filter paper. Let the medial surface of the hemispheres dry for a few seconds (Figure 2G). **Note: Leaving the brain on the filter paper for too long will make it hard to remove it.** Next, transfer each hemisphere using the square spatula from the filter paper onto the glue on the vibratome plate in the same orientation (the medial side of the hemisphere should be glued to the base plate). **Critical: Avoid touching the glue with the bottom side of the spatula to prevent it from inadvertently sticking, which will spoil the setting of the brain properly.** Using another flat spatula to lightly push the brain onto the glue could help.
    - Place the vibratome plate into the vibratome chamber and secure the chamber with the designated screw to the vibratome, so that the cut posterior surface faces the blades. **Note: Depending on what plate you use, you may need to plan ahead the angle at which you mount the brain so that you are able to fasten the plate inside the slicing chamber without the cut-out part** (crescent shape in Figure 2H) **facing one of the fastening screws.** Use the remaining sucrose solution in the syringe and 50 mL cup to gently cover, wet, and cool the glued brain. Fill the vibratome chamber with slushy sucrose from the 250 mL plastic cup, until both hemispheres are entirely submerged in the solution. **Note: Pour sucrose only around the chamber perimeter and not straight onto the hemispheres to avoid injuring them.**
    - Set the vibratome’s speed to 3.6 on the dial, slice thickness to 275 µm, and frequency to (9–10) on the dial. Additionally, set the start and end points of the blade in each cycle. Make sure to set them so the blade will cut through the entire hemisphere each time (consult the VT1000 S manual).
*Note: Slice thickness may change depending on which brain region is being studied.*
    - To obtain sagittal slices of the striatum (which is what we are describing in this protocol), discard the first few slices that include only cortical tissue. Once the blade is medial enough to produce slices in which the striatum can be seen, begin collecting them using a Pasteur pipette and transfer them individually to the slice chamber. There are typically six slices until you reach the anterior commissure, at which point you should stop collecting slices. The Pasteur pipette can also be used with gentle presses to move ice chunks away from the space between the blade and the brain.
    - Wait for at least 60 min from the moment all relevant slices are collected before beginning electrophysiological experiments to allow the tissue to recover from the slicing and to accommodate to the physiological conditions created by aCSF after being submerged in sucrose solution.
- **Patch-clamp recording**
  1. Patch-clamp recordings are used in all the experiments in which we want to test how blue LED activation of the proximal vs. the entire somatodendritic arbors affects the currents recorded in the soma. As we are interested in the effect of dendrites on synaptic inputs, these experiments are conducted while holding the soma at subthreshold voltages (the choice of voltage can affect the results [15]). The relevant blue LED perturbations for these experiments include brief pulses (see E below), Poisson barrages of brief pulses (see G below), or the sequence of sinusoids of various frequencies (see F below).
  2. Flowing solutions into the recording chamber (at a rate that should be between 1 and 5 mL/min; slower than that may not be sufficient to provide the oxygen and nutrients to the slices, and faster than that can make the recording unstable due to movement of the slice):
    - Prepare the K internal and aCSF (with the necessary synaptic blockers, see Reagents) in advance, adjusting their concentrations as required.
    - Load the K internal solution into a 1 mL syringe affixed to a 0.22 μm syringe filter and a microloader, maintaining it at a cold temperature on ice.
    - Initiate a 20 min flow of double-distilled water prior to the experiment to ensure the cleanliness of the entire setup.
    - Continuously oxygenate the aCSF with carbogen for 20 min preceding the experiment. Then, 5 min prior to commencing the experiment, initiate a flow of oxygenated aCSF into the recording chamber to maintain the physiological conditions.
  3. Float the anti-vibration optical table.
  4. Fire up electrophysiology setup:
    - Turn on micromanipulator and temperature controllers, A/D board, amplifier, LED, microscope lamp, and PC computer.
*Note: Make sure the amplifier and computer are grounded to avoid electrical noise.*
    - Set the temperature controller to 31 °C.
*Note: This is a value that we find as a good compromise between keeping the cell healthy for a longer time (the cells deteriorate more rapidly at higher physiological temperatures) and reproducing more physiologically relevant autonomous firing rates than observed at room temperature.*
      Activate software in the following order: Camera software, Multiclamp commander, and Signal 6.
      **Figure 3.**
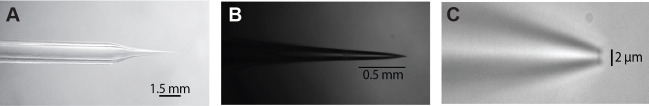
Patch pipette dimensions. The patch pipette has to be pulled so that it has a shaft (A) that is long enough to fit under the hi-mag objective and has nice taper (B) that ends with a 1–3 µm opening (C).
  5. Prepare microelectrodes with micropipette puller from glass pipettes, adjusting their resistance to the desired range, typically between 4 and 5 MΩ. (Consult puller manual on how to pull microelectrodes; see Figure 3.)
  6. One hour after the incubation period, carefully transfer the sagittal brain slice to a Zeiss Axioskop fixed-stage microscope and secure it using a U-shaped harp.
  7. Locating cell to patch:
    - Use the lo-mag objective lens (5×) to locate the light source positioned beneath the brain slice, which can be identified visually.
    - Switch to the hi-mag objective lens (60×) in order to visually identify the ChR2-expressing neuron of interest. Note: We have studied striatal cholinergic interneurons (CINs) in ChAT-ChR2 mice and GABAergic projection neurons in the substantia nigra pars reticulata (SNr) in Thy1-ChR2 mice. Once a healthy neuron has been found, situate it in the center of the screen.
    - Subsequently, load the microelectrode with the K internal solution and meticulously eliminate any air bubbles, particularly at the tip. **Caution: Return to the low-magnification objective lens to prevent any inadvertent damage to the pipette.**
    - Affix the loaded microelectrode securely to the microelectrode holder and take caution to lock the holder in place, thus maintaining a stable setup.
  8. Patching the cell:
    - Before lowering the microelectrode into the recording chamber, exert slight positive pressure to prevent any potential filth from entering the pipette tip.
*Note: Use a manometer to set the correct pressure. The manometer should be connected with a T-junction to the tube used to pressurize the pipette through its connection to the microelectrode holder. It is useful to use a 3-way Luer Lock on the end of the tube used by the experimenter to control, manipulate, and stabilize the pressure.*
    - Precisely position the pipette tip at the center of the field of view using the lo-mag objective lens.
    - Use the micromanipulators to carefully lower the microelectrode into the recording chamber.
    - The amplifier should be in voltage clamp (VC) mode. Conduct a seal test to assess the pipette's resistance. *Note: If the resistance measures too low, it may indicate a compromised pipette tip, whereas excessively high resistance may suggest blockage, either due to debris or a wrong/faulty puller program.*
    - Switch back to the hi-mag objective lens, submerging it into the bath (usually, by gradually moving upwards) while searching for the pipette tip. Be cautious to prevent inadvertent pipette tip breakage during this process.
    - When initiating the micropipette descent toward the tissue, after locating its tip, proceed with caution in a downward motion. **Caution: Always move the objective lens first, followed by *catching up* with the micropipette, so you can see the new region before moving the pipette there, so as to prevent inadvertently crashing the pipette into the slice**. Commence this procedure at an elevated velocity (using one of the C settings on the L&N manipulator controller), while continuously monitoring the resistance of the micropipette.
    - Once you visualize the tissue, move to a lower speed (H, M, or L on the L&N controller) to minimize potential tissue damage. Lower only the objective to verify presence of the cell in the same initial location. Subsequently, raise the objective back up to the micropipette and methodically continue the downward progression towards the cell; again, first the objective and then the pipette.
    - Position the tip of the pipette at the optimal viewing point for observing the cell, ensuring that all boundaries are within view, rendering the cell at its maximal observable size.
    - Delicately approach the cell surface. When done properly, this will result in an increase of micropipette resistance (by approximately 0.2 MΩ) and the appearance of a discernible "dimple" between the cell and the micropipette, signifying close contact between the micropipette tip and the cell surface.
**Critical: At this point, adjust the pipette offset via the MultiClamp 700B commander to minimize the voltage offset.**
    - Subsequently, release the positive pressure and gently apply a continuous negative pressure through oral suction, causing a notable increase in the seal resistance, ultimately reaching >1 GΩ. This is called the *cell-attached* mode.
    - While the cell is approaching the gigaseal, adjust the holding voltage to the desired holding membrane potential (which should equal the cells’ expected resting membrane potential or, for pacemaker neurons like CINs or SNr neurons, to a voltage close to their zero current, which is typically at -60 mV) using the holding button in the MultiClamp 700B commander.
    - To prevent saturating the amplifier, leading to distortions in the measured currents, conduct capacitance compensation (in MultiClamp commander, press the auto button opposite *Cp fast* and *Cp slow*, which will compensate for the fast and slow transients. It may be necessary to adjust *Cp slow* manually).
    - Following stable seal formation, carefully apply an additional slight negative pressure through the pipette to break-in the cell membrane to obtain a *whole-cell* recording. After break-in, access resistance should ideally be low (typically < 10 MΩ). If the access resistance is too high, then the membrane is not completely broken, and gentle negative pressure can correct this. For *Cp fast* and *Cp slow*, optimal values can vary, but *Cp fast* should typically be in the 2–5 pF range and *Cp slow* between 10 and 30 pF (depending on the size of the neuron).
    - Improve voltage-clamp performance through the compensation of series resistance (Rs). (In MultiClamp commander by activating the Rs compensation button. Strive to reach a correction factor of 75%.)
*Note: Rs compensation does not always work.*
    - In whole-cell mode, we use VC mode to measure currents and current clamp mode to measure voltage.
  9. In the experiments described below, we compare *proximal* to *full-field* optogenetic stimulation. In the proximal illumination, we strive to activate ChR2 in a small region (approximately 100 µm in diameter, a size that is determined by the smallest pin-hole we could produce in the disk placed near the back focal plane of the objective and used to block out most of the field of view) around the soma, nominally activating the soma and proximal dendrites. In the full-field stimulation, we strive to activate the entire somatodendritic field of the neuron. **Note: The optogenetic stimulation could activate neighboring neurons that innervate the patched cell. It is, therefore, critical to include the appropriate synaptic blockers in the recording chamber aCSF solution to ensure that measured effects are postsynaptic.** See also General Notes.
- **Perforated patch recording**
  1. The perforated patch configuration is used to study the effect of the blue LED light perturbations on the firing pattern of the pacemaking neuron. Here, we use current clamp in order to record how the input affects the timing of the autonomous action potentials of the pacemaking neuron. The relevant perturbations are: a) long sinusoidal waveforms set to frequencies below, in the vicinity of or above the autonomous firing rate of the pacemaker neuron (see H below), and b) the barrage (see G below) that can be used to estimate both the phase response curve (PRC) of the neuron [21–24] and the peri-stimulus time histogram (PSTH) of the neuron in response to a sudden increase in ChR2 currents
  2. The perforated patch method is a variation of patch clamp that establishes electrical access between the cell and the patch pipette using pore-forming antibiotics (here, we use Gramicidin). The resulting pores selectively permit small monovalent ions while preserving the integrity of many cytoplasmic components and the autonomous spiking of pacemaking neurons.
  3. To prepare the Gramicidin internal, first add 5 mg of Gramicidin into 500 μL of anhydrous Gramicidin in DMSO. This stock solution can be stored at room temperature and used for three days, after which it should be discarded.
  4. Prefilter 1 mL of K internal solution by drawing it into a syringe affixed to a syringe filter. Combine 1–2 μL of the stock solution with 1 mL of internal solution, resulting in a final concentration of 10–20 μg/mL. Note: This quantity has to be determined individually based on experience and practice attaining a seal. *Front-fill* the recording pipette with regular K internal by connecting the pipette's back to a syringe using soft tubing, immersing the tip in K internal solution, and applying negative pressure with the syringe. Note: We prefer to use pipettes without a filament (to reduce capillary action), but other groups do not. Fill 2/3 of the tip with regular K internal solution. Use a microloader to backfill the pipette with Gramicidin internal to the standard level.
  5. For successful perforated patch recordings, it is crucial to establish a gigaseal while the pipette tip still has clean K internal solution. This will prevent Gramicidin from spewing onto the cell surface during the patching process. To prevent expelling all clean K internal solution before reaching the cell, which can interfere with the formation of a seal, apply very low positive pressure (<0.5 psi) until the pipette is just above the slice, increasing it immediately before entering the slice. Speed is also crucial for achieving this goal, with the pipette tip ideally positioned above the cell within one minute of entering the bath. Note: The final concentration of Gramicidin can also be titrated on the fly during the experimental day by adjusting the volume of backfilled Gramicidin internal to speed up or slow down the perforation.
  6. While speed is essential, it is equally vital to be gentle; minimal dimpling of the cell membrane before releasing pressure to create the gigaseal typically yields more stable recordings.
  7. After a gigaseal has been established, perforation usually takes 10–30 min to complete (at 31 °C). We judge the extent of the perforation by the size of the action currents in voltage clamp mode that should be >300 pA in amplitude or the size of the action potentials in current clamp that should overshoot -20 mV (but preferably 0 mV).
- **Switching apertures for *proximal* vs. *full-field* optogenetic illumination**
  1. Since at this time point, after completion of the patching, you are using the hi-mag objective lens, you begin with arranging the proximal 470 nm LED illumination (Figure 4A, red region) by sliding in the IRDIC analyzer fitted with the disk with the small hole into the analyzer slot just behind the back of the objective, and closing the diaphragm of the incident-light pathway (Figure 5). Later, after the protocols are run in the proximal configuration, we then open the diaphragm, pull out the analyzer with the disk, pull the water immersion high-magnification objective out, and swing in the low-magnification air/dry objective for the full-field illumination (Figure 4A, green region).
    **Figure 4.**
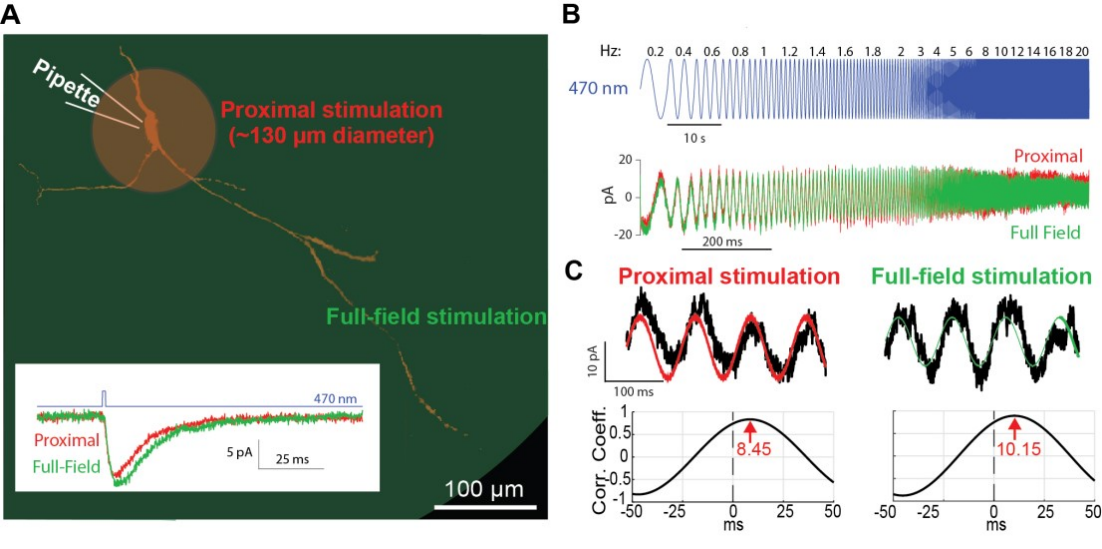
Optogenetic activation of proximal vs. full-field dendritic arbor. A. Schematic of the proximal (red) vs. full-field regions of illumination. Inset: decay time of current response to a brief pulse of blue light illumination is longer for full-field (green) than proximal (red) illumination. B. Concatenated sinusoidal waveforms of blue light illumination (top) are used to elicit current responses to either full-field (green) or proximal (red) illuminations (bottom). C. The input waveforms (red or green) are cross-correlated with the current (black) response (top) to generate the respective cross-correlograms (bottom). The latency of the peak (in seconds) is translated into a phase by multiplying it by the driving frequency.
    **Figure 5.**
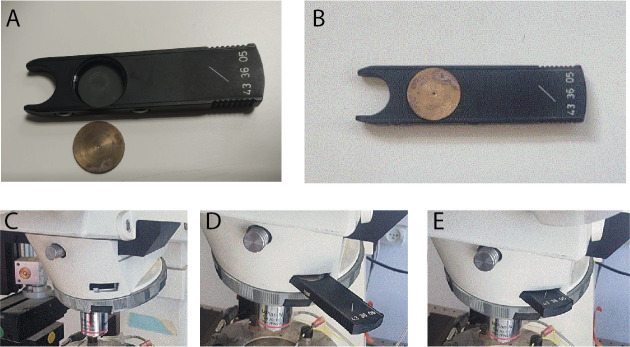
Proximal illumination. A. A disk, with a central pinhole, is machined to fit into the Axioskop analyzer. B. The disk is fitted into the analyzer. C. The analyzer slot in the incident 470 nm light pathway is situated near the back focal plane of the objective lens. D. The analyzer with the disk is gently inserted into the slot. E. The disk with the central pinhole is in place near the back focal plane of the objective. Note: Disregard the lo-mag air objective in the images; proximal illumination is done via the high-mag water-immersion objective.
- **Brief 470 nm pulse perturbation**
  1. In voltage clamp mode, while holding at a hyperpolarized voltage (e.g., -70 mV), apply a brief 1-ms-long blue light pulse to elicit an inward current. Repeat several times to extract the average response (Figure 4A, inset).
  2. The ChR2 is expressed in the postsynaptic neuron that you are interrogating, and you are therefore stimulating it directly. Nevertheless, you could be activating synaptic inputs into the neuron (e.g., collaterals of the neuron you are stimulating or of other neuronal types that express ChR2 in the slice). Thus, it is pertinent to include synaptic blockers in the aCSF that are relevant in the neuronal type you are studying [we commonly use blockers of ionotropic glutamate (10 mM DNQX & 50 mM D-AP5), GABA_A_ (10 mM gabazine), and GABA_B_ (2 mM CGP55845) receptor blockers].
  3. Later, after completing all the *proximal measurements*, repeat in the full-field illumination configuration. **Critical: The amplitude of the LED voltage should be chosen (by trial and error) so that the currents generated in the somatic recording pipette will be on the order of approximately 10 pA. This parameter needs to be set separately for each of the illumination configurations. The amplitudes required for the proximal illumination are typically larger by an order of magnitude, because most of the light is blocked by the disk making the effective illumination small.**
- **Sinusoidal 470 nm perturbations**
  1. Program (in advance) a whole-cell voltage clamp protocol in Signal 6 in which the soma is held at a subthreshold voltage and in which the 470 nm LED’s analog driver is fed a waveform that is composed of concatenated sinusoids (Figure 4B). **Critical: The amplitude of the LED voltage sinusoidal should be chosen (by trial and error) so that the currents generated in the somatic recording pipette will be on the order of a few tens of picoamperes.** The frequencies should include both sub- and supra-Hertz values and should be chosen so that each frequency is represented by an integer multiple of cycles so that the overall waveform will be continuous. We used the following frequencies (durations indicated in parentheses): 0.2 Hz (5 s), 0.4 Hz (5 s), 0.6 Hz (5 s), 0.8 Hz (5 s), 1 Hz (5 s), 1.2 Hz (5 s), 1.4 Hz (5 s), 1.6 Hz (5 s), 1.8 Hz (5 s), 2 Hz (3 s), 3 Hz (3 s), 4 Hz (3 s), 5 Hz (3 s), 6 Hz (3 s), 8 Hz (3 s), 10 Hz (3 s), 12 Hz (3 s), 14 Hz (3 s), 16 Hz (3 s), 18 Hz (3 s), and 20 Hz (3 s). Note: In Signal 6, you can actually program this long waveform using their built-in protocols. Alternatively, you can program the waveform with another software and upload it into a Signal 6 protocol. The sampling frequency of the voltage signal needs to not be too high because these are low-frequency sinusoidals (we used 10 kHz).
  2. Optional: Repeat the stimulation several times to average the response and reduce noise. The waveform can be inverted in time and fed into LED to control history dependence.
  3. In order to study the dependence of the results on holding potential, steps F1–2 can be repeated at various holding potentials (e.g., -70, -60 & -50 mV).
  4. Later, after completing all the proximal measurements, repeat in the full-field illumination configuration.
- **Barrage 470 nm stimulation**
  1. Program (in advance) 25 individual realizations of a Poisson barrage of brief (0.5–1 ms) 470 nm LED pulses using interpulse intervals (IPIs) that are distributed exponentially (Figure 6C) [13]. Note: The barrage waveforms will need to be generated with another software (e.g., MATLAB) and uploaded into a Signal 6 protocol. The mean IPI is a parameter to be chosen depending on the neuron’s spontaneous firing rates (the idea is to get several dozen pulses between each spike). The amplitude of the pulses needs to be set to affect the spike timing without dramatically altering the ongoing firing rate. This intensity will be different for proximal vs. full-field illumination.
    **Figure 6.**
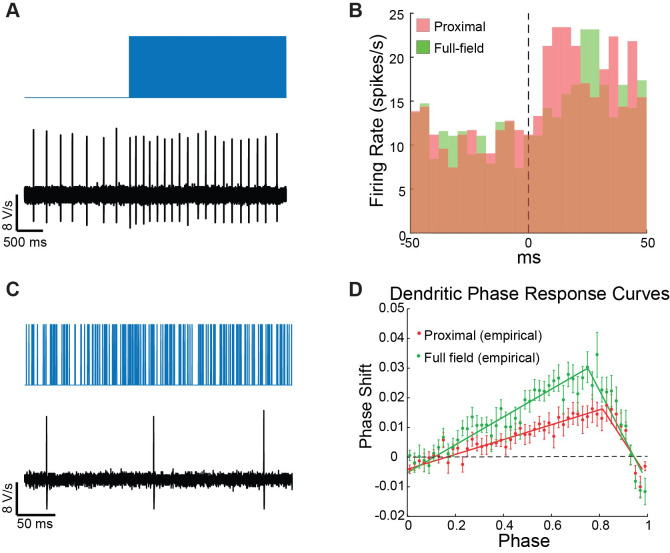
Poisson Barrage of blue light pulses is used to estimate the neuron’s peristimulus time histogram (PSTH) and dendritic phase response curve (dPRC). A. On the large time scale, the barrage acts as a current step function, which drives an increase in the pacemaker neuron’s firing rate. B. The PSTHs for proximal (red) and distal illuminations (green) are estimated by binning the spiking response over multiple trials. C. At a finer time scale, dozens of individual pulses are visible between spikes. D. The sequences of pulses leading up to each spike are used to estimate the dPRC via regression analysis.
  2. After patching the cell, allow the perforation to advance (this can be done either while holding the cell in voltage clamp and watching the action currents gradually increase, or in current clamp—the spike amplitude increases to approximately 0 mV).
*Note: This experiment can also be conducted in cell-attached mode. If so, we suggest holding the cell in current clamp, because in voltage clamp you will inadvertently be clamping the cell as it perforates.*
  3. Once perforated, run the 25 realizations of the Poisson process and record the spiking.
  4. As above (step E2), include synaptic blockers.
  5. Later, after completing all the *proximal measurements*, repeat in the full-field illumination configuration, with the required changes in barrage parameters (mostly intensity).
- **Oscillatory entrainment in perforated patch**
  1. After establishing a gigaseal, allow the perforation to advance (as described in step C7 above).
  2. Estimate the autonomous firing rate of the neuron.
  3. Program 30–60 s long protocols on Signal 6 (on the fly) of 470 nm LED sinusoids with frequencies that are below, above, and near the autonomous rate (you can also try integer multiples of the autonomous rate) and run these (Figure 7A). Note: The aim is to use 470 nm LED intensities (that will vary for proximal vs. full-field illuminations) that will slightly perturb. Trial and error is inevitable. Additionally, the frequency of the pacemaking neuron may change during the course of the experiment, so estimate it frequently and adapt.
    **Figure 7.**
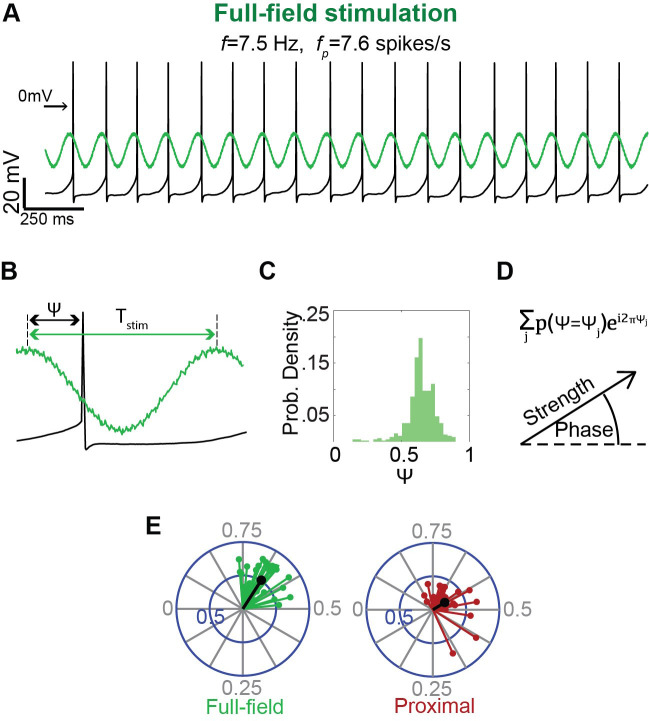
Estimation of the degree and phase of entrainment of a pacemaker neuron to oscillatory inputs. A. A long sinusoidally modulated blue light waveform, at a frequency (f) near the autonomous frequency (fp) of the pacemaker, is used to entrain the pacemaker neuron (recorded in the perforated patch configuration) either via full-field (left) or proximal (right) illumination. B. Estimation of the phase of the input 𝜓 at which the spike occurs (we defined the phase of the trough as phase 0 and the peak as phase 0.5). C. Histogram of the phases 𝜓 at which the neuron fires, exhibiting a narrow peak indicative of entrainment. D. Histogram used as a probability distribution function P(𝜓) from which the circular variance vector is calculated using the displayed formula. The modulus of the resulting complex number is used to estimate the strength of the circular variance vector, which is used, in turn, to determine whether entrainment is significant (as compared to threshold determined by bootstrapping). The phase of entrainment is extracted from the argument of the complex number (divided by 2𝜓). E. An example of the distribution of significant circular variance vectors estimated for full-field (green) vs. proximal (red) illumination.

## Data analysis

- **Dendritic phase and amplitude estimation and model fitting**
  1. Estimate phase and amplitude response at each driving frequency *f*.
    - Parse the long current trace to segments corresponding to the LED driving at each frequency *f* (Figure 4C).
    - Calculate the cross-correlation function (CCF) between each segment and the sinusoidal waveform used to drive the LED using the MATLAB function xcov with the parameter SCALEOPT set to ‘biased’(Figure 4C).
    - Locate the peak of the CCF using the MATLAB function findpeaks with the parameter MinPeakHeight set to 0 (Figure 4C). **Note: You may need to visually inspect the CCF to verify the identification of the peak as the one closest to the zero.** Convert the location of this peak to units of seconds and then multiply time by the driving frequency *f* to convert to phase.
    - Find the maximal value of the CCF function.
*Note: Normally, this corresponds to the value of the CCF at the peak you found in c above. However, in some cases the maximal deflection from the zero of the CCF amplitude is a negative peak, and then the amplitude should be taken from that. Divide this value by the amplitude of the voltage sinusoidal used to drive the LED to get a value in units of pA.*
  2. You will typically need to repeat this experiment on at least three animals and get a sufficient quantity of cells (in the order of 10 or so), in order to extract a mean and standard error of the mean (SEM) for the phase and amplitude. From our experience, the error on the estimation of the phase is very small, whereas the error on amplitude is very large.
  3. There are qualitative findings you can extract from the data at this point. You might see a change in the amplitude of the response in the presence of a drug or at a different holding potential [15], or a difference in the phase response between proximal and wide-field stimulation [14,15]. These findings are more robust in the sense that they are model-independent.
  4. Fitting a model to the data enables you first to fit specific parameters to the data and, perhaps more importantly, to rule out models when the points cannot be fit by them. The best example of this is the appearance of a negative peak in the phase response, which can only be explained by the presence of a restorative current such as the hyperpolarization-activated cyclic nucleotide-gated (HCN) current.
  5. **Note: The derivation of the various models is beyond the scope of this *bio-protocol*.** You are referred to the original papers in which we used these protocols [14,15] for the various models, which are based on the assumption that the amplitude and phase responses are the concatenation of two filters: one arising from the filtering properties of the ChR2 current response and the other arising from the contribution of dendritic and somatic passive membrane properties and nonlinear conductances. The ChR2 current filters because it deactivates with particular time constants. The somatodendritic arbor filters because of the passive and semi-active membrane properties that arise from its specific capacitance, leak channels, and nonlinear ionic channels. We developed equations for these filters in previous publications (under the assumption of a very simple geometry of a dendrite). You are encouraged to refer to those [9,14,15] to understand the derivations and the assumptions they are based on.
  6. Models are fit with the lsqcurvefit function in MATLAB. Note: In principle, you should be able to fit the parameter by simultaneously fitting the mean amplitude values and the mean phase values. However, on a practical level, we found that first fitting the parameters to the phase model (whose error bars are much tighter) and then using the extracted parameters to fit the additional parameter of the overall single scale of the amplitude response worked more robustly.
- **Dendritic peristimulus time histogram (PSTH) estimation**
  1. The PSTH we refer to is measuring how the neuron’s firing rate changes in response to a step function increase in current and should be estimated for neurons recorded either in perforated patch or cell-attached mode (which does not disturb the neurons intracellular milieu). While you can add a separate protocol for this purpose, this will require many more measurements. Instead, we found that using the barrage stimulus suffices, because on the time scale of changes in firing rate, the barrages behave like a step-function increase in activation (Figure 6A).
  2. Generating a PSTH requires many action potentials. Depending on the rate of the neuron, this will typically require many trials per neuron and pooling many neurons (for SNr neurons, it was on the order of 20 neurons, see Figure 4 in Tiroshi et al [14]).
  3. Extract the spike times used in units of seconds for each of the trials.
  4. The PSTH can be calculated using the MATLAB command histc, which returns a count of spikes that fall within each bin. To convert this to an instantaneous firing rate, you need to divide this number by the number of trials and by the width of the bin used in the histc command (Figure 6B).
*Note: You will probably need to try several bin widths until you find one that gives the best looking rendition.*
- **Dendritic phase response curve (dPRC) estimation**
  1. The dPRC depicts how the pacemaker neuron’s phase is influenced by small voltage perturbations delivered to the dendrite as a function of the timing of the perturbation [9]. dPRCs are estimated based on slight modifications to previously published methods [13,25].
  2. Detect spike times using the MATLAB function findpeaks with the parameter MinPeakHeight set to the average of the minimal and maximal values.
  3. Divide each interspike interval (ISI) into 50 equally sized bins (i.e., for each ISI, the bin size scales with the duration of the ISI), where the *j*th bin corresponds to the phase *φ_j_ = (j-0.5)/50*.
  4. Count the pulses delivered in each bin and denote the number of pulses delivered in the *α*th ISI and *j*th bin by n*p_α_*,*_j_*. Calculate the mean number of pulses per bin, averaged across all bins, and subtract it from *p_α_*,*_j_* resulting in *Δp_α_*,*_j_*.
  5. Perform a multiple regression analysis with *Δp_α_*,*_j_* values as the independent variables and ISI durations as the dependent variables. If we denote the PRC as *Z(φ)*, then the regression coefficients *Z(φ_j_)* provide a unique solution for the PRC (Figure 6D, Netoff et al., 2011).
- **Measuring entrainment**
  1. The degree of entrainment of the cell’s spiking activity to rhythmic inputs is evaluated based on previously described methods [13]. Throughout the analysis, we consider the effective phase ψ of each spike, which is its phase with respect to the period *T* of the rhythmic stimulation (Figure 7B).
  2. To quantify the strength of entrainment:
    - For each cell and each stimulation frequency, plot the distribution of effective phases estimating *Pr(ψ = ψ_j_)* for each phase *ψ_j_* (Figure 7C).
    - Use this estimation to generate a circular variance vector, which provides a measure of the variation of effective phases:
$$\sum_{j}Pr(\psi =\psi_{j})e^{2\pi i\psi_{j}}$$
    The amplitude of the circular variance vector represents the extent to which the firing of the neuron was entrained by the oscillatory input, and its phase indicates the effective phase of entrainment, which corresponds to the peak of the distribution of effective phases (Figure 7D,E).
  3. In order to investigate phases of entrainment, only include trials where the spiking of the neuron was successfully entrained:
    - Use bootstrapping to generate a distribution of the amplitude of circular variances. In each bootstrapping iteration, the surrogate data should consist of a series of *N* uniformly distributed random numbers (between 0 and 1), where *N* is the typical number of ISIs in a trial.
    - Only trials where the amplitude of the circular variance is larger than the 95^th^ percentile of the bootstrapping distribution, calculated as described previously [14], should be included in the analysis.

## Validation of protocol

This protocol or parts of it has been used and validated in the following research article(s):

- Tiroshi L et al. (2019). Population dynamics and entrainment of basal ganglia pacemakers are shaped by their dendritic arbors. *PLoS Computational Biology* (Figure 2–6).
- Oz O et al. (2022) Non-uniform distribution of dendritic nonlinearities differentially engages thalamostriatal and corticostriatal inputs onto cholinergic interneurons. *eLife* (Figure 1–3)

## General notes and troubleshooting

**General notes**

- Some laboratories perfuse intracardially with aCSF (i.e., not with modified aCSF), while other laboratories skip the perfusion altogether and decapitate the deeply anesthetized animal. In our hands, and particularly when working with neurons we study (e.g., striatal cholinergic interneurons, basal ganglia pacemaker neurons) the perfusion with sucrose yields slices that are healthier for longer.
- Optogenetic stimulation, particularly in the full-field condition, might elicit activation of nearby neurons, some of which may project to the patched neuron. To ensure that the measured effects are generated post-synaptically, the aCSF solution should include antagonists for the channels expressed by the neuron. For our recordings in the striatum and substantia nigra, we supplemented the aCSF with DNQX to block AMPA receptors, D-AP5 to block NMDA receptors, SR to block G_ABAA_ receptors, and CGP to block G_ABAB_ receptors. Different blockers may be necessary for experiments conducted in other brain regions.
- The 470 nm waveforms used throughout the protocol must have a minimal value at the threshold voltage of the LED, i.e., approximately 40 mV.
